# Policy Implications of Achievement Testing Using Multilevel Models: The Case of Brazilian Elementary Schools

**DOI:** 10.3389/fpsyg.2016.01727

**Published:** 2016-11-23

**Authors:** Igor G. Menezes, Victor R. Duran, Euclides J. Mendonça Filho, Tainã J. Veloso, Stella M. S. Sarmento, Christine L. Paget, Kai Ruggeri

**Affiliations:** ^1^Laboratory of Quantitative, Methods for Predictive Psychometrics, Psychology Institute, Federal University of BahiaSalvador, Brazil; ^2^Judge Business School, University of CambridgeCambridge, UK; ^3^Department of Education, University of OxfordOxford, UK; ^4^Policy Research Group, Department of Psychology, University of CambridgeCambridge, UK

**Keywords:** large-scale educational assessment, school achievement, hierarchical linear modeling, education policies, Brazilian education system

## Abstract

Large-scale educational assessment has been established as source of descriptive, evaluative and interpretative information that influence educational policies worldwide throughout the last third of the twentieth century. In the 1990s the Brazilian Ministry of Education developed the National Basic Education Assessment System (SAEB) that regularly measures management, resource and contextual school features and academic achievement in public and private institutions. In 2005, after significant piloting and review of the SAEB, a new sampling strategy was taken and Prova Brasil became the new instrument used by the Ministry to assess skills in Portuguese (reading comprehension) and Mathematics (problem solving), as well as collecting contextual information concerning the school, principal, teacher, and the students. This study aims to identify which variables are predictors of academic achievement of fifth grade students on Prova Brasil. Across a large sample of students, multilevel models tested a large number of variables relevant to student achievement. This approach uncovered critical variables not commonly seen as significant in light of other achievement determinants, including student habits, teacher ethnicity, and school technological resources. As such, this approach demonstrates the value of MLM to appropriately nuanced educational policies that reflect critical influences on student achievement. Its implications for wider application for psychology studies that may have relevant impacts for policy are also discussed.

## Introduction

In Latin America, most educational assessment systems were established during the 1990s, either as an unfolding of accumulated experience in assessments for specific purposes or as part of education reform programs that were common in that decade. Those national systems vary largely in terms of their institutional framework; technical, financial, and operational provisions, as well as the social, economic, and political ambiance in which they operate. Economic fluctuations and political instability affect national educational assessment systems differently, and it has been recognized that those systems supported by institutions that are independent of education ministries—and thus enjoy greater technical and management autonomy—tend to be more stable (Ferrer, 2006). That is the case of Brazil's National Institute of Educational Studies and Research (Instituto Nacional de Estudos e Pesquisas Educacionais Anísio Teixeira–INEP).

Created in the late 1930s as a research and consulting agency to support the Brazilian educational system, INEP began to play a strategic role in the 1990s, as the education reform established systematic, comprehensive, large-scale educational assessments with increasingly important effects over educational policies in the following decades. It's relative independence and accumulated experience warranted INEP to consolidate and refine techniques that now directly influence all agents involved in the teaching-learning process that takes place within the scope of the national education system: government, schools, teachers, parents, and students.

After 21 years of military government, the process known in Brazil as “re-democratization” entailed a comprehensive reform of the state, initiated by the review and enactment of a new Federal Constitution in 1988. The state reform was first operationalized in the health sector, with the establishment of a universal, integral and decentralized Unified Health System (SUS) by national law in 1990. In the same year the National System for Educational Assessment (*Sistema de Avaliação da Educação Básica*-SAEB) was first administered by INEP to a sample of public schools in 25 Brazilian states to produce in-depth understandings regarding the outcomes of teaching and learning processes in the national education system (Ministério da Educação, 2001).

Under the National Educational Bases and Guidelines Law (*Lei de Diretrizes e Bases da Educação*-LDB) in 1996, SAEB gained a strategic role in guiding policies aimed at enhancing the quality of education in Brazil. Following provisions of the new Constitution, LDB established not only a universal and decentralized educational system, but was also required to develop a National Plan for Education that would set forth guidelines, goals and strategies for educational policy every 10 years (Castro, 1998; Dourado, 2002; Castro and Menezes, 2003).

SAEB underwent important transformations in each subsequent edition (1997, 2001, 2003), comprising technical improvements (e.g., creation of Test Specifications, since there was no National Curriculum in Brazil at that time) and broadening of the scope of information collected to cover more academic subjects and aspects of school and family context. In 2005, SAEB went on to encompass two major assessments: the National Basic Education Assessment (*Avaliação Nacional da Educação Básica*—ANEB), that addressed management aspects and was sample based, and the National Student Achievement Assessment (*Avaliação Nacional do Rendimento Escolar*—ANRESC) or *Prova Brasil*. *Prova Brasil* benefited from successive improvements to its SAEB predecessor and, by being census and not sample based, allowed the creation of achievement indicators for individual schools, municipalities, cities, states, and Brazil as a whole. Therefore, *Prova Brasil* introduced accountability into Brazil's educational scenario.

*Prova Brasil* measures skills in Portuguese—with focus on reading comprehension—and Mathematics—with focus on problem solving. Students also complete a questionnaire designed to collect sociodemographic and family information, as well as school records and habits (studying habits, culture seeking, and so forth). Teachers and school principals participate in data collection by completing questionnaires regarding school infrastructure and working conditions, as well as personal and professional background information.

With *Prova Brasil*, national, regional, and local statistics are now being generated, which allow direct comparisons that provide the government with relevant information that could be used for the development and monitoring of educational public policies. The Basic Education Development Index (Í*ndice de Desenvolvimento da Educação Básica*—IDEB), launched in 2007, introduced a sophisticated informational tool to guide political action by combining proficiency indicators from *Prova Brasil* with information about students (approval) into a single scale that ranges from 0 to 10. IDEB facilitated the monitoring of schools with low student achievement and, as an accountability device, was used as criterion for providing technical and financial resources based on these schools' development plans (Napier, 2014).

One of the goals of the National Education Plan (*Plano Nacional de Educação*—PNE) for 2011–2020 was for the IDEB to reach a national score of 6 by 2021, which would place Brazil at the same level of current academic achievement of developed countries that are members of the Organization for Economic Cooperation and Development–OECD. The current national public system IDEB score, calculated in 2013, was 4.9 in the early years of elementary school (0.2 above what was projected) and 4.0 in the final years (0.1 under projection, Ministério da Educação, 2015).

Brazil's public educational expenditure rose from 3.5% of GDP in 2000 to 6.1% in 2011, which represented the highest growth of investment in education of any OECD and G20 partner countries (OECD, 2014a). In the elementary education system, most of this investment is directed toward the enhancement of educational through additional resources to schools, especially technology, infrastructure, and teacher training. For Brazil to achieve these development goals, policies must be developed with greater precision, a task that requires a substantial understanding of the interactions, potential, and limitations of a variety of factors in a multilevel structure. Educational effectiveness studies are made complex by the mesh of micro factors that interact at each level of education (Cervini, 2002) however, the search the most important variables at each level must continue as must their role in the development of educational policies (Cervini, 2006).

## Materials and methods

### Participants

The total number of participants for Mathematics was 7726 schools, 11,421 classes and 127,256 students, and for Portuguese was 7688 schools, 11,361 classes, and 126,527 students. With regard to the total amount of observations after listwise deletion, most of the sample was composed by females (*M* = 51.4%, *P* = 51.4%). Students were mainly from public schools (*M* = 81.2%, *P* = 81.2%), and the 10-year-old group was the most frequent (*M* = 54.3%, *P* = 54.2%). The majority of students were from the Southeast (*M* = 60.4%, *P* = 60.6%), followed by the South (*M* = 15.6%, *P* = 15.2%), Northeast (*M* = 14.5%, *P* = 14.5%), North (*M* = 5.5%, *P* = 5.5%), and Midwest (*M* = 4.1%, *P* = 4.3%) regions. Although the relative frequencies for the sample differ from that ones observed for the population, the rank order remains the same, i.e., in the population, the Southeast was the region with more students answering *Prova Brasil*, the same occurring in the sample. The same pattern was repeated for all regions.

### Instruments

#### Variable selection

According to Ferrão (2003), variables associated with both internal and external factors affecting school must be taken into account when assessing achievement. Thus, based on the literature and previous studies conducted in Brazil that investigated the relationships between academic achievement and student sociodemographic variables, this research selected a set of 22 relevant variables to control for their effects. To achieve a better understanding of the variables investigated in this study, they are explored here at each of their levels. The variables for the first level (students) are: sociodemographic characteristics, cultural and social capital, motivation and self-esteem, studying habits and school records (Albernaz et al., 2002; Jesus and Laros, 2004; Menezes-Filho, 2007; Brasil, Ministério da Educação, 2008; Riani and Rios-Neto, 2008; Castro, 2009; Couri, 2010). For the second level (classes), the following teacher variables were selected: sociodemographic characteristics, level of education and expectations, professional experience, pedagogical practices, and working conditions (Mello, 1994; Ferrão and Fernandes, 2001; Albernaz et al., 2002; Soares and Alves, 2003; Menezes-Filho, 2007; Riani and Rios-Neto, 2008; Castro, 2009; Felicio, 2010; Palermo, 2011). Finally, the third level (school characteristics) was comprised of the sociodemographic characteristics of the principals, level of education and expectations, professional experience, leadership, working conditions of staff, collaborative work, academic and disciplinary practices, pedagogical resources and school facilities and equipment (Jesus and Laros, 2004; Soares, 2004, 2007; Andrade and Laros, 2007; Biondi and Freitas, 2007; Menezes-Filho, 2007; Riani and Rios-Neto, 2008; Castro, 2009; Castelar et al., 2012; Passador et al., 2012; Lamas et al., 2013).

The variable selection procedure was carried out based on a theoretical framework study conducted by Franco et al. (2003). This work was based on previous systematic reviews and made relevant contributions to the theoretical and empirical field of educational assessment in Brazil. SAEB survey questionnaires were developed in conformity with this seminal research, which ultimately guided the variable selection for *Prova Brasil* questionnaires. Constructs and variables for each level can be shown in Table 1.

**Table 1:** **Levels and their selected variables for predicting academic achievement in *Prova Brasil***.

**Level**	**Group**	**Variables**
First	Socio demographic characteristics	Age, ethnicity, gender, parent education, socioeconomic status
	Cultural and social capital	Culture seeking behaviors, study habits, reading habits, hours dedicated to household chores, child labor
	Parents	Parents involvement
	School records	School dropout, school failure
Second	Sociodemographic characteristics of teachers	Teacher's ethnicity, gender
	Teacher's level of education and expectations	Teacher's level of basic and supplementary education
	Pedagogical practices	General pedagogic practices
	Working conditions	Number of schools the teacher works, contract type, working hours per week
Third	Staff working conditions	Actions to prevent violence, violence inside school
	Academic and disciplinary practices	Presence of school dropout program, supporting program for students
	School facilities and equipment	Information and communications technology, public areas

This study aims to identify how much of the variance in academic achievement of fifth grade students on *Prova Brasil* is explained by each one of the three levels (students, classes, and schools), and which of the aforementioned variables are the best predictors of academic achievement.

The variables presented in this study are those deemed statistically significant for both Mathematics and Portuguese. Also, these variables are the ones that have the largest chi-square, corroborating the decision above.

This study used data from five different instruments. Four questionnaires are designed to assess sociodemographic characteristics of schools, teachers, and students. The fifth measure is *Prova Brasil* itself. The students' questionnaire has 54 items concerning sociodemographic background characteristics and also the engagement of students and their parents in education-related activities. This questionnaire is completed by students right after *Prova Brasil* has been administered. The teachers' questionnaire is comprised of 119 items concerning their background, pedagogical practices, and their socioeconomic and cultural profile. It also contains items regarding the classes attending the exam, such as availability of essential academic resources. Each school is assessed by two separate instruments; the first has 72 items and focuses on the characteristics of the school. The second has 212 items and is also completed by the school principal, who reports data related to school infrastructure, resources and available materials, school safety and school conservation status, as well as the practices and attitudes of teachers and students. Lastly, *Prova Brasil* has two parts of 22 items each for Portuguese and Mathematics, based on specifications of the Brazilian National Education Curriculum. Prova Brasil scores are based on the SAEB proficiency scales, which split the achievement levels into different categories regarding distinct levels of cognitive ability. The Portuguese scale is comprised of nine categories, with scores ranging from 0 to 350. The Mathematics scale is bit larger and is divided in 12 categories, ranging from 0 to 425.

### Data analysis procedures

#### Dataset preparation

This study utilizes secondary datasets obtained from the Brazilian Ministry of Education website (Brasil, Ministério da Educação, 2015). Every student, teacher, principal, and school has a unique identification number which permitted a merge of all of the datasets into two matrices, one containing achievement information for Portuguese and the other for Mathematics. Producing separate datasets is justified because different sociodemographic information is available for each area of knowledge, both for students and teachers. Additionally, the main goal during the merging process was to maximize the number of observations with complete data (i.e., without missing cases).

The rate of missing data for all instruments is high, which is aggravated by the hierarchical structure of the data. That is, missing information about a single school is propagated to sometimes hundreds of students that belong to that same school. We believe that the length of the questionnaires is a possible explanation for the high frequency of missing data. The voluntary, low-stakes nature of the Prova Brasil testing is also likely a factor in the large amount of missing data (see Sundre and Wise, 2003; Wise and DeMars, 2005, for example).

The first stage of the merging process joined all datasets into a single one without removing any observation, generating entire rows of missing data from unmatched information across the datasets. The second step was to remove all variables not to be considered in the hierarchical model. Finally, all observations with missing values were excluded using listwise deletion, reducing the amount of observations from an initial 5.2 million to 127,256 and 126,527 observations for Mathematics and Portuguese, respectively. These procedures aimed not only to minimize lost information but also to preserve group structure, which is essential to perform Multilevel Modeling adequately. Although, it is acknowledged that this listwise deletion strategy has limitations (Little and Rubin, 2002), such as massive losses of data that could increase the probability of Type II errors (King et al., 2001), Allison (2002) states that listwise deletion will produce unbiased estimates even if the data are not missing at random. Furthermore, the author points out that listwise deletion might reasonably be preferred over maximum likelihood or multiple imputation if a large amount of data remains after the deletion procedure.

**Figure 1:**
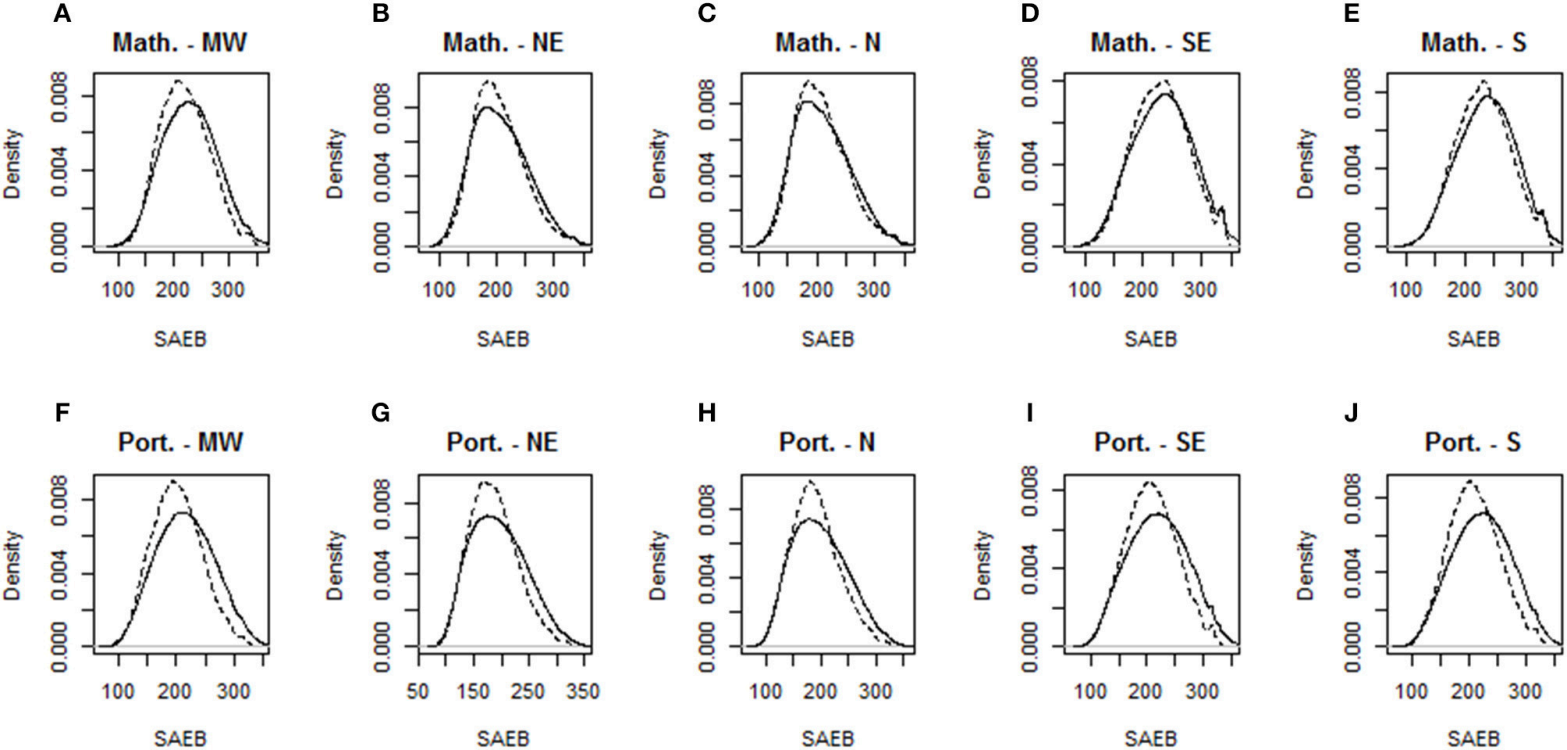
**Density functions comparing achievement between population and sample for Portuguese and Mathematics by regions**. MW, Middle West; NE, Northeast; N, North; SE, Southeast; S, South.

In order to compare for differences in the distributions of the population and the sample designed for this study, density functions were calculated and plotted. In general, no major disparities were observed, as can be seen in Figure 1.

#### Multilevel modeling

When investigating critical factors—whether common or those less regularly considered—there are considerable challenges to appropriately analyse the available data. This requires a hierarchical approach, which takes into account potential conflicts in variation within and between classrooms and schools. As such, it is argued that the multilevel model approach is best suited to address these in a robust and appropriately nuanced way, considering the many potential levels of impact relevant to effective educational policy. MLM is a generalization of linear regression models particularly suited for hierarchically structured data, wherein information is repeated for multiple observations that belong to the same group. With improvements in computer performance observed in the late 1980s, Multilevel Modeling (MLM) started to be widely used in education as a technique for the prediction of academic achievement. Three hierarchies, also known as levels, were defined for *Prova Brasil*: students, classes and schools. The information available for a given school is repeated across all students of that school, which is ideally modeled by a MLM. This phenomenon contributes to increasing collinearity among covariates in the data, which, in standard regression settings, produce biased estimates. The chosen MLM resolves this issue by estimating a separate intercept for each group in higher leveled covariates, which, in turn, reduces the bias in the slope terms (Gelman and Hill, 2007).

Academic achievement measures the extent to which students perform in different academic subjects. For this study, it is set as the dependent variable, and the variables investigated at the three levels of the MLM are the independent ones. Two types of models were tested in this research. The first one was a *null model*, which does not consider the independent variables at any of its levels and therefore does not explain any variance in the dependent variable; it only estimates the partitioning variance of the dependent variable due to each of the levels considered. The second was a *conditional model*, which is drawn from the null model, with the independent variables for each level being added in order to explain part of the total variability.

For both null and conditional models, only random intercepts were considered in this study. In effect, for a three-level null model with random intercepts, the student's achievement (*Y*_*ijk*_) is predicted from the sum of the average of their class (β_0*jk*_) with the error (*e*_*ijk*_), which represents the distance between their score and the average of their class. In addition, the student's class average will be predicted at Level 2, from the student's school average (γ_00*k*_) plus a random effect (*u*_0*jk*_). Finally, the student's school average will be predicted at the Level 3, from the student's school overall average (π_000_) plus a random effect (*r*_00*k*_). All random effects are assumed to be normally distributed, with mean equals zero, and variance σ^2^. The three-level null model with random intercept is shown in Equation (1).

(1) $$\begin{array}{l} Y_{ijk}=\beta_{0jk}+e_{ijk}\\ \beta_{0jk}=\gamma_{00k}\;+\;u_{0jk}\\ \gamma_{00k}=\pi_{000}+r_{00k} \end{array}$$

Where,

*i* = 1, 2, 3, …, *i* students in the *j*^*th*^ class of the *k*^*th*^school
*Y*_*ijk*_: academic achievement of the student *i* studying in the class *j* of the *k* school
β_0*jk*_: academic achievement mean score for the class *j* of the school *k*
*e*_*ijk*_: random error of the *i*^*th*^ student in the *j*^*th*^ class of the *k*^*th*^school
γ_00*k*_: academic achievement mean score in school *k*
*u*_0*jk*_: random error of the *j*^*th*^ class for the *k*^*th*^ school
π_000_: overall average, considering the total of students
*r*_00*k*_: random error of the *k*^*th*^ school

In order to identify predictive sociodemographic variables for academic achievement in Prova Brasil, this study utilized two types of MLM models: a null and a conditional model, as defined at the introduction.

The procedures for fitting the multilevel model were as follows:

- To estimate a Null-Model for the two two-level models—students and classes, and students and schools—, and for the three-level model in order to check the variance partitioning among the levels for each model;
- To compare the goodness-of-fit between each null-model and select the model that best fits the data and explains the most variability found at the student level. Three indices were calculated for that: (a) The log-likelihood ratio, which tests the hypothesis that the variance in achievement between subjects on the same level is zero. Also, it assumes the existence of effects of higher levels within the individual level. Log-likelihood statistic is calculated using an adaptive Gauss-Hermite approximation; (b) The Akaike's Information Criterion (AIC), which compares the models being tested to a hypothesized true distribution. The lower the AIC value, the better the model; and (c) BIC, Bayesian Information Criterion, which can be interpreted in the same fashion as the AIC, but the fitted models are based on the posterior probability distribution. The more probable the model, the more defensible it is;
- To investigate whether the residuals follow a normal distribution. Moreover, both class and school effects must also be significantly different from zero;
- To estimate three different random intercept conditional models considering the selected variables for each level using the Restricted Maximum Likelihood method;
- To calculate the three statistics introduced at the second step for each conditional model in order to compare the deviance between the models. Again, the smaller the model deviance in comparison to others, the better its fit;
- For each variable to be considered in each conditional model, the following statistics are produced: Chi-square, the regression Beta, and a t estimate. The Chi-square is interpreted as a deviation of group means from the grand mean and it gives the test statistic for each variable in the model. The Betas have the same interpretation as in standard regression, the average increase in the dependent variable given a one point increase in the predictor. The t statistic is used to obtain a *p*-value which assess the significance of the variable to the model as a whole.

## Results and discussion

### Descriptive statistics

Mean scores for Portuguese Language and Mathematics are presented in Table 2.

**Table 2:** **Mean and standard deviation scores at Prova Brasil 2011-Portuguese and Mathematics (SAEB scale)**.

**Dataset**	**Mean SAEB scores**					
	**MW *M (SD)***	**NE *M (SD)***	**N *M (SD)***	**SE *M (SD)***	**S *M (SD)***	**General *M (SD)***
Population	228.53	206.08	208.72	234.10	238.45	224.52
Math.	(48.27)	(48.16)	(47.55)	(50.77)	(48.67)	(51.12)
Population	216.31	195.01	198.32	219.92	222.77	211.45
Port.	(50.39)	(50.33)	(50.08)	(53.10)	(50.70)	(52.90)
Sample	219.50	202.45	206.02	230.32	232.57	224.85
Math.	(42.98)	(43.31)	(43.75)	(46.60)	(45.63)	(46.99)
Sample	201.12	185.48	189.01	207.70	208.71	203.33
Port.	(42.72)	(42.80)	(42.53)	(45.39)	(43.66)	(45.31)

MW, Midwest. NE, Northeast; N, North; SE, South East; S, South.

### Model selection

The first stage of the model selection procedure consisted of assessing the influence of higher levels on individual scores based on statistics of likelihood ratio and explained variance. This analysis guided the decision concerning which levels would be included in the MLM. A total of three models were tested, with all combinations of models containing two and three levels.

Although, the variability of academic achievement accounted for by the 2 two-level models tested are similar ($\sigma_{classes}^{2}M=24.77\%$ and $\sigma_{classes}^{2}P\;=$ 18.62% for classes and $\sigma_{schools}^{2}M=23.65\%$ and $\sigma_{schools}^{2}P\;=$ 17.25% for schools), in the three-level model the variance is proportionally higher for schools than for classes ($\sigma_{classes}^{2}M=6.33\%$ and $\sigma_{classes}^{2}P=5.97\%$ for classes and $\sigma_{schools}^{2}M=18.89\%$ and $\sigma_{schools}^{2}P=12.86\%$). This suggests that the variance proportion due to differences between schools should be taken into consideration in multilevel modeling and the three-level model will therefore be more likely to better explain academic achievement. In spite of that, a log-likelihood ratio for each null-model was calculated so as to verify what model contains less deviance. The log-likelihood is calculated using an adaptive Gauss-Hermite approximation. The test assesses the existence of effects of higher levels within the individual level.

Both class and school effects were significantly different from zero, with a 95% confidence interval estimated from the residuals estimated at levels two and three respectively. The ML deviance for the two-level model (students and schools) for Mathematics was 1322197 (AIC: 1322203; BIC: 1322233), whereas the ML deviance for the three-level model (students, classes and schools—Mathematics) was 1321171 [AIC: 1321179; BIC: 1321218; *p* < 0.001; $\Delta {\text{X}}_{\left(1\right)}^{2}=1026.8$], showing therefore a better fit for this model. Likewise, the ML deviance for the two-level model for Portuguese was 1312406 (AIC: 1312412; BIC: 1312442), whereas the ML deviance for the three-level model was 1311610 [AIC: 1311618; BIC: 1311657; *p* < 0.001; $\Delta {\text{X}}_{\left(1\right)}^{2}=$ 796.51], showing then a better fit.

The chosen model for both disciplines included higher levels for class and school. The results of the likelihood ratio tests were significant, which suggests that the three-level model is significantly different from the others, justifying the increase in complexity. However, as with all tests of the chi-squared family, large sample sizes produce tests that are too powerful, which increases the chances of type I errors. The specificities of the chosen conditional model are described below.

### Three-level model

Once the multilevel model that best explains academic achievement was fitted, a set of variables from each of the three levels was chosen based on the significance of their chi-square, slope of the regression line (Beta), associated t-statistic and respective *p*-values. Table 3 shows the estimates calculated for each variable deemed important to represent its respective level ordered by their chi-square in Portuguese. For Portuguese, 15 variables were selected to represent the first level (student), 11 variables to the second level (teacher/classes), and 11 variables to the third level (schools). For Mathematics, 15 variables were selected to represent the first level (student), 11 variables to the second level (classes), and 10 variables to the third level (schools). Analyses of the results in light of the Brazilian education policies addressing the topics are provided for variables with highest statistical significance and beta, separated by level.

**Table 3:** **Standardized coefficients, chi-square, and significance levels of the first level variables**.

**Variable**	**Category**	**Portuguese**			**Mathematics**		
		**χ^2^**	**β**	***t***	**χ^2^**	**β**	***t***
Culture seeking behaviors	Numerical	4540.5430^***^	−8.63	−67.38	3432.62^***^	−7.59	−58.58
School Failure	Once	2888.01^***^	−19.06	−53.02	2760.45^***^	−18.93	−51.93
Baseline: Never	Twice or more		−15.22	−25.89		−14.82	−24.91
Study habits:	Sometimes	1919.29^***^	7.81	10.49	2367.83^***^	6.83	7.61
Baseline: Never or hardly ever	Always or frequently		18.71	25.61		20.22	22.98
Hours dedicated to household chores	1 h	1380.70^***^	−0.90	−3.05	1529.43^***^	1.34	4.47
Baseline: Zero	2 h		−4.89	−13.57		−2.77	−7.60
	3 h		−9.57	−21.09		−8.41	−18.36
	4 h or more		−14.63	−29.36		−14.12	−28.02
Reading Habits	Numerical	1232.01^***^	4.54	35.10	580.47^***^	3.14	24.09
Baseline: Zero							
Gender	Female	1181.30^***^	7.77	34.32	1316.72^***^	−8.27	−36.28
Baseline: Male							
Mother Education	Does not Know	868.05^***^	2.36	2.76	868.05^***^	2.57	2.97
Baseline: illiterate	Primary school until 4th year		0.77	0.86		3.11	3.44
	Primary school until 8th year		3.98	4.58		5.04	5.73
	Incomplete high school		4.74	5.37		6.47	7.25
	Incomplete undergraduate degree		12.54	14.11		12.23	15.53
	Undergraduate degree		5.51	6.05		4.82	6.04
Ethnicity	Asian	713.77^***^	−1.79	−2.08	694.16^***^	−0.05	−0.06
Baseline: White	Black		−9.15	−20.27		−10.24	−22.45
	Brazilian Indian		1.41	1.77		−0.68	−0.85
	Brown		−3.02	−11.94		−2.50	−9.77
Child Labor	Does not know		−8.45	−20.13		−7.37	−17.38
Baseline: Does not work	Work	710.35^***^	−10.10	−26.65	347.96^***^	−7.15	−18.65
Father Education	Does not Know	494.55^***^	4.36	6.08	465.71^***^	5.73	7.89
Baseline: illiterate	Primary school until 4th year		0.97	1.24		4.57	5.75
	Primary school until 8th year		5.98	7.88		7.85	10.20
	Incomplete high school		3.33	4.35		4.72	6.08
	Incomplete undergraduate degree		10.86	13.96		12.23	15.53
	Undergraduate degree		3.65	4.62		4.82	6.04
Socioeconomic Status	Numerical	450.65^***^	2.75	21.22	893.08^***^	3.95	29.88
Parent involvement	Numerical	336.11^***^	2.90	18.33	109.12^***^	1.20	10.44
Student's age	9 years	200.73^***^	12.13	1.91	283.64^***^	18.93	2.90
Baseline: eight years or less	10 years		16.24	2.57		23.19	3.56
	11 years		15.94	2.52		21.99	3.38
	12 years		11.33	1.78		16.42	2.51
	13 years		10.27	1.61		15.74	2.40
	14 year		9.11	1.42		16.00	2.43
	15 years or more		11.64	1.80		13.07	1.96
School dropout	Once	19.19^***^	−0.62	−1.03	11.33^**^	−0.32	−0.52
Baseline: Never	Twice or more		−4.98	−4.30		−3.87	−3.34

Significance levels:

*** p < 0.001;

** p < 0.01;

^*^ p < 0.05.

### First level

The variables that most strongly predicted change in scores of *Prova Brasil* were the same for Portuguese and Mathematics at the first (student) level, as displayed on Table 3.

Variables associated with greater increase in scores for both tests were, in order of magnitude: study habits, student's age, mother, and father education and gender. Parent involvement, reading habits and socioeconomic status (SES) were also associated with significant positive variation in Portuguese and Math scores.

Variables most significantly associated with decrease in Portuguese and Math achievement as measured by *Prova Brasil* were school failure, amount of time dedicated to household chores, child labor, ethnicity, school dropout, and culture seeking behaviors.

In this study, study habits corresponded to the frequency with which students did homework. Always or frequently doing homework was the variable that most positively impacted both Portuguese and Math achievement, followed closely by student's age, which is an age-within-grade indicator.

Evidence on the impact of homework on school achievement has been accumulated for almost a century. Although, there is consensus on the positive impact of homework, many factors must be considered, such as educational level, academic subject, time spent on homework, to what extent the teachers correct, feedback, and fit homework to content explored in class (Núñez et al., 2015). Many studies show that impact of homework on achievement varies between educational levels, being neutral or negative in some grades of Elementary Education and more positive in High School (Cooper et al., 2006). Murillo and Martinez-Garrido (2014) found that assigning homework is a common practice in Latin America, although correcting homework or using it in teaching sessions is not so frequent, especially in Brazil, when compared to 15 other latin american countries. In our study, doing homework frequently exerted a positive impact over Brazilian 5th graders' Math and Portuguese achievement, regardless of whether or not it was supported by efficient use in classroom.

Being 10 and 11 years old—thus having the expected age for 5th grade in Brazil (Ministério da Educação, 2009)—predicted a large amount of positive variation over both Math and Portuguese scores. Being 9 years old (expected age at the beginning of 4rth grade) was the third greatest variation on Portuguese and Math achievement as accounted for by student's age, which tended to decrease between 12 and 14 years. Research on the relation between age and achievement show that years of schooling may exert a greater influence over academic achievement than years of age (Cliffordson and Gustafsson, 2010). Martin et al. (2011) advert that age-within-grade variation is largely influenced by national educational policies regarding age of entry to school and promotion/retention. The authors indicate that countries that are more flexible regarding age of school entry and that use retention practices are those with greater age variation within a certain grade, as our shown by our results. By analyzing data of 4th graders on the PIRLS 2006 countries (that would correspond to 5th graders in Brazil), Martin et al. (2011) found that in those countries with strict age cutoffs for entering school and policies of automatic promotion, older students showed higher average reading achievement when compared to the younger students. Nevertheless, PIRLS 2006 countries with a lesser degree of economic development tended to have the oldest students at 4th grade and the lowest average reading achievement.

Higher mother and father education, parent involvement and higher family socioeconomic status (SES) had positive effects, consistent with many other studies such as Hattie's (2009) meta-analysis of 800 other meta analyses related to school achievement in English speaking, highly developed countries. The author found that family SES, parental education (what can be considered part of SES indicator) and parental involvement have medium to large effect sizes over student achievement (0.57, 0.60, and 0.51 respectively). Many studies also show to what extent ethnicity and father's occupation are significant contributors to student achievement (Peng and Hall, 1995; McCoy, 2005). Parental education and family SES level show positive correlations with the student's quality of achievement (Parelius and Parelius, 1987; Caldas and Bankston, 1997; Ma and Klinger, 2000; Mitchell and Collom, 2001; Jeynes, 2002). Students with high level of SES perform better than middle class students and middle class students perform better than students with low level of SES (Garzon, 2006; Kahlenberg, 2006; Kirkup, 2008). The effect of a student's SES on their academic achievement is widely accepted in both international educational literature (Gray et al., 1990; Mortimore, 1991; Goldstein et al., 1993; Sammons, 1995) and Brazilian literature (Hasenbalg and Valle Silva, 1990; Harbison and Hanushek, 1992; Souza and Valle Silva, 1994; Arias et al., 2004; Fernandes, 2004).

Student's gender had significant impact for both Portuguese and Mathematics. Results showed that female 5th graders performed better in Portuguese and worse in Mathematics. The relationship between gender and academic achievement has been discussed for decades (Eitle, 2005). A gap between the achievement of boys and girls has been found, with girls showing better achievement than boys in certain disciplines (Chambers and Schreiber, 2004). In a meta analyses that used 100 sources with 259 independent effect sizes suggested that gender differences in Mathematics achievement are small and differences favoring men emerge in high school and in college (Hyde et al., 1990). In a more recent study (Lindberg et al., 2010) gender difference ranged between −0.15 and +0.22 (negative values indicating superior achievement by females). Supporting the view that males and females perform similarly in mathematics, although larger positive values were found at the male group. Similarly, a meta-analysis study with scholastic achievement found a female advantage for language courses (Voyer and Voyer, 2014).

The variable that predicted the greatest amount of decrease on academic achievement at the first level was school failure. Another outcome frequently related to school failure—school dropout—showed a lower but significant negative effect over 5th graders achievement both in Portuguese and Math in our study. These results suggest a shortcoming in Brazilian education policy because students that fail attend the same classes in the next year with no adaptation to the program aimed to deal with student's individual deficiencies (de Leon and Menezes-Filho, 2001). Motivation is also an important element to be considered (Fortier et al., 1995). Low motivation may be related to school failure and bad school achievement, establishing a vicious circle.

It is important to highlight that LDB established that Elementary Education in Brazil *might* be organized in grades or cycles (among other options), the latter implying continuous progression and reduction of school failure. Therefore, each state Education Department is free to adopt a modality—either organizing Elementary Education in cycles, solely in grades, or a combined model. As discussed previously, Brazil's Education System is decentralized and encompasses three levels (city, state, and union), each with its own subsystems in each level of education. Currently, only four Brazilian states adopt grades for the whole range of Elementary Education—the other 20 states and the Federal District adopt cycles for at least the first years of schooling, and four of them adopt cycles throughout the level. The 2013 School Census revealed that 21.3% of primary schools in Brazil were organized in cycles (Ministério da Educação, 2014). This rate meets UNESCO's “Education for all in 2015” (United Nations Educational Scientific and Cultural Organization–UNESCO, 2007) recommendation to introduce continuous progression within cycles in over 10% of schools in Brazil, thereby reducing failure and retention—and our results support the findings that indicate the detrimental effect of failure on school achievement among 5th graders.

Nevertheless, continuous progression divides opinions between educators and experts in Brazil, especially because implementation of the strategy is not always accompanied by recommended educational practices such as reinforcement and recovery activities, alternative means and adaptation, teachers training, and therefore likely (Jacomini, 2004; Neves and Boruchovitch, 2004); results in a condition labeled “automatic promotion” (Gadotti, 2003) usually associated with low academic achievement. Menezes-Filho et al. (2009) compared achievement in *Prova Brasil* 2005 by graded school students vs. students of schools that adopted cycles (therefore continued progression). The study showed that the effect of cycles is significant in reducing dropout rates for all levels of education. On the other hand, the effect of continued progression was not significant on 5th graders' achievement in *Prova Brasil* 2005, and had a negative and significant impact on the achievement of 9th graders. A study commissioned in São Paulo, the first state to adopt cycles in full length in Elementary Education, consulted teachers, parents and students from all state regions and found that continuous progressions was the second most important problem faced by public schools, according to teachers. Teachers, parents and students agreed that security issues/violence was the most important problem (APEOESP, 2014).

Students extensively engaged in household chores and work related activities performed worse than the other students. These findings corroborate a previous study by Alberto et al. (2011) who found that children and adolescent workers have lower school attendance, with consistently poor consequences in learning. Apart from worse achievement in standardized tests, children living under impoverished conditions face a higher level of stress, affecting their memory, learning, and achievement. Moreover, they have higher rates of morbidity and chronic diseases, and more emotional and behavior problems (Moore et al., 2009; Lefmann and Combs-Orme, 2014).

For instance, in 1988 when the new Federal Constitution was enacted, about 15% of 7–14 year old Brazilians were not enrolled in school. The last national census, carried out in 2010, showed a significant decrease in out-of-school children/adolescents rate, reaching 3.3%. The most affected by exclusion were the 4–5 and 15–17 age groups. Reasons for being out of school were similar throughout age groups and mirrored the country's grave social inequalities—the majority were poor, afro-descendant children from parents or guardians with little or no education, living in rural areas (UNICEF, 2014). In accordance with these findings, school failure, ethnicity (afro-descendants) and child labor were the most detrimental factors on 5th grade student achievement as measured by *Prova Brasil* 2011.

Student's culture seeking behaviors (attending museums, cinemas, theaters) showed a negative relationship with student's achievement in Mathematics and Portuguese. These results contradict (Gaddis, 2013) investigation in which typical operationalizations of cultural capital (high-arts participation and reading habits) has positive effects on grade point average. However, these findings might be due to type of cultural habits experienced by students since it was not possible to assess the qualitative aspects of this variable, for instance the kind of books read or movies seen.

### Second level

All the details of the variables at the second level (Class) and their relationship with academic achievement are displayed in Table 4.

**Table 4:** **Standardized coefficients, chi-square, and significance levels of the second level variables**.

**Variable**	**Category**	**Portuguese Language**			**Mathematics**		
		**χ^2^**	**β**	***t***	**χ^2^**	**β**	***t***
Teacher's ethnicity	Does not know	59.77^***^	−3.04	−1.33	113.99^***^	−3.68	−1.42
Baseline: White	Asian		−1.49	−1.18		−1.22	−0.86
	Black		−1.66	−1.18		−3.10	−4.23
	Brazilian Indian		−5.11	−2.42		−6.47	−2.68
	Brown		−2.87	−7.40		−4.51	−10.30
Teacher working hours per week	20 h per week	48.91^***^	−0.64	−0.39	53.64^***^	−1.19	−0.63
Baseline: Less than	21–24 h per week		4.12	2.31		5.80	2.87
	25 h		−0.18	−0.10		−0.01	−0.01
	26–30 h per week		1.94	0.98		1.40	0.62
	30 h per week		−0.74	−0.44		−1.30	−0.67
	31–39 h per week		2.10	1.23		2.98	1.54
	40 h per week		2.14	1.34		1.04	0.58
	More than 40 h per week		2.42	1.51		1.03	0.57
General pedagogic practice	Numerical	35.14^***^	1.07	5.92	35.44^***^	1.21	5.95
Teacher Gender	Female	18.14^***^	2.56	4.25	14.37^***^	2.48	3.79
Baseline: Male							
Teacher's level of education	High school	16.89^***^	−1.04	−0.18	17.86^***^	1.93	0.30
Baseline: elementary school	Completed university		2.31	0.40		5.73	0.90
Number of schools the teacher works	Two	12.18^***^	−1.47	−3.39	12.18^***^	−1.68	−3.42
Baseline: One	Three		−0.71	−0.63		−1.73	−1.38
	Four or more		0.40	0.21		−3.02	−1.43
Teacher's contract type	Temporary (no formal contract)	9.50^*^	−4.31	−2.19	18.09^**^	−3.33	−1.47
Baseline: Statutory	Temporary (with formal contract)		−1.04	−1.92		−2.05	−3.38
	CLT (consolidation of labor laws)		0.34	0.60		1.02	1.58

Significance levels:

*** p < 0.001;

** p < 0.01;

* *p < 0.05*.

The first two variables to have a significant relationship with academic achievement in Mathematics and Portuguese are teacher ethnicity [$\chi_{\left(M\right)}^{2}$ = 113.99, *p* < 0.001; $\chi_{\left(P\right)}^{2}$ = 59.77, *p* < 0.001] and gender [$\chi_{\left(M\right)}^{2}$ = 14.37, *p* < 0.001; $\chi_{\left(P\right)}^{2}$ = 18.14, *p* < 0.001]. The results indicate that better achievement is obtained when teachers are white and male.

Discussions about the attempt to explain differences in achievement for Portuguese and Mathematics tasks must take into account the influence of teacher's characteristics. According to Dee (2007), some theories suggest that many of these differences are related to the teacher's gender. It is believed that students were more well behaved and performed better when taught by a teacher of the same gender. Conversely, Antecol et al. (2015) bring a review about findings concerning the impact of teachers on different academic results, as well as teacher's gender influence on students from primary education. Their results showed that female teachers have a negative influence to scores of elementary school students in mathematics compared to male teachers.

Empirical evidence resulting from research in this field, as showed by Escardibul and Mora (2013), point out that an accurate determination of the relationship between gender of teachers and student achievement in primary and secondary education is yet to be established. It is prudent to consider that our objective was to analyze the best hierarchical model that may substantiate political policies aiming to improve the achievement of students in Portuguese and Mathematics, and not focused on exploring the determinants of gender influence of teachers on the achievement of students.

Regarding the ethnicity variable, according to Dee (2004), some theories suggest that ethnic interaction between teachers and students can influence the achievement of those in various ways. Students tend to trust and respect those who share certain characteristics, making learning easier. Also a teacher of the same ethnic group can be seen as an effective role model, increasing confidence and enthusiasm for learning. Conversely, there is little direct empirical evidence to support these claims. This study linked students with teachers of the same ethnicity (i.e., afrodescendant teachers with black students, and so on), which proved to be influential in the achievement of students. However, it was thought a posteriori that these differences could be attributed to not directly observed qualitative differences.

A possible explanation for our results indicating that white teachers have a positive influence student achievement may be related to better access to education and qualification for whites as opposed to other ethnicities. Thus, having better educational opportunities, white teachers might develop a better technical training which may have contributed to the observed result.

The amount of time that the teacher worked per week [$\chi_{\left(M\right)}^{2}$ = 53.64, *p* < 0.001; $\chi_{\left(P\right)}^{2}$ = 48.91, *p* < 0.001] was also significantly associated with student achievement. The worst performing group in both subjects were those students taught by teachers who worked “30 h per week.” The structure of the response options themselves must be addressed, since the choice of defining some categories as intervals and others as a single value might add unaccounted bias to the results. Moreover, the item also referred to the total amount of time devoted exclusively to teaching, not accounting for administrative work or time spent preparing classes.

As discussed by Arelaro et al. (2014) there are different types of contracts for teachers in Brazil. In most, hours spent in extracurricular activities are assumed to be part of the workload, and we cannot know if they were considered by teachers as working hours when answering the question. The authors also state that in most schools the “teaching hour” is equivalent to a 45-min class, which would mean that a 30 h a week contract implies in 22.5 “in-class” teaching hours. Again, this uncertainty was likely transferred to the data, adding noise to the observed results. This might explain the lack of a clear trend concerning the different levels of the weekly workload variable. Further assessment of the interactions between this variable and the number of schools a given teacher works at might clarify the relationship between worked hours and achievement.

The variable that accounts for the number of schools a teacher works is directly related to the number of hours and displays a more interpretable trend [$\chi_{\left(M\right)}^{2}$ = 12.18, *p* < 0.001; $\chi_{\left(P\right)}^{2}$ = 12.18, *p* < 0.001]. With the exception of Portuguese teachers working at four or more schools, working in more schools was associated with worse student achievement. This pattern can be explained by increased stress and less time available for class preparation and further qualification of the teacher (Skaalvik and Skaalvik, 2011).

The diversity of pedagogic practices used by the teacher was also deemed important [$\chi_{\left(M\right)}^{2}$ = 35.44, *p* < 0.001; $\chi_{\left(P\right)}^{2}$ = 35.14, *p* < 0.001]. The variable assessed whether a teacher uses in-class aids, such as computers, the internet or comic books. The relationship was positive, which means that better achievement was expected from those students taught by teachers who incorporated learning aids. Recent developments in the literature support these findings, and further states that the effects of learning aids can be particularly effective for students with lower achievement (Sung et al., 2015). The authors also report improvement in pupils' attitudes toward the subject after contact with such practices. Attitudes were not assessed directly by SAEB but could also positively influence achievement in the long term (Chou et al., 2015).

The influence of the level of qualification of a teacher was also relevant to achievement [$\chi_{\left(M\right)}^{2}$ = 17.86, *p* < 0.001; $\chi_{\left(P\right)}^{2}$ = 16.89, *p* < 0.001]. There is a general positive trend in achievement for students of increasingly qualified teachers, with some unexpected deviations from said trend. One exception was a lower average score in Portuguese students of teachers who graduated high school when compared to those who studied up to elementary school only. Since the requirements for teacher qualification increased with time in Brazil this may be explained by the amount of years of experience those older teachers have compensating for the lack of formal education, although that would require further investigation to confirm. This difference is not observed in Mathematics, which could be associated with the modernization of teaching techniques, as discussed by Fiorentini (2005), that could outweigh the influence of experience.

### Third level

All the details of the variables at the third level (School) and their relationship with academic achievement are displayed in Table 5.

**Table 5:** **Standardized coefficients, chi-square, and significance levels of third level variables**.

		**Portuguese language**			**Mathematics**		
**Variable**	**Category**	**χ^2^**	**β**	**T**	**χ^2^**	**β**	**t**
Information and communications technology	Numerical	75.26^***^	2.06	8.67	90.86^***^	2.62	9.53
Presence of school dropout program	Yes	46.68^***^	−2.89	−6.83	61.32^***^	−3.87	−7.83
Baseline: None							
Public Areas	Numerical	25.83^***^	1.08	5.08	28.18^***^	1.32	5.30
School food service	Numerical	23.30^***^	1.02	4.82	39.71^***^	1.56	6.30
Violence inside school	Numerical	17.58^***^	−1.09	−4.19	20.69^***^	−1.38	−4.19
Presence of school supporting program for students	Yes	14.13^***^	2.53	3.76	30.30^***^	4.28	5.50
Baseline: None							
Actions to prevent violence	Numerical	12.00^***^	0.69	3.46	13.91^***^	0.87	3.73

Significance levels:

*** p < 0.001;

^**^ p < 0.01;

^*^ p < 0.05.

The school level variables that most impacted achievement were that ones related to national programmes aimed at the reduction of school dropout [$\chi_{\left(M\right)}^{2}$ = 61.32, *p* < 0.001; $\chi_{\left(P\right)}^{2}$ = 46.68, *p* < 0.001] and school support programmes focused on tutoring and learning difficulties monitoring [$\chi_{\left(M\right)}^{2}$ = 30.30, *p* < 0.001; $\chi_{\left(P\right)}^{2}$ = 14.13, *p* < 0.001]. Over the last two decades, far-reaching welfare measures and social programmes were developed to eradicate extreme poverty, most of which have contributed significantly to preventing school dropout and supporting families who consistently send their children to school. Some national programs, such as Brazil's National School Feeding Programme and *Bolsa Familia* have also helped reduce the school dropout rate. According to the INEP *Bolsa Familia* alone has been responsible for a decrease of as much as 36% of school dropout rates.

The presence of regional and national school support programs has led *Prova Brasil* scores to increase significantly for both Portuguese and Mathematics. Most of these programs are directly associated with investments in information/communication technology, which have also provided small, but significant improvements to the student's achievement [$\chi_{\left(M\right)}^{2}$ = 90.86, *p* < 0.001; $\chi_{\left(P\right)}^{2}$ = 75.26, *p* < 0.001]. The National Program of Educational Technology (ProInfo) is one of the most important policies focused on promoting the educational use of information technology by students in public schools. Federal government provides states or municipalities with the equipment needed to set up computer labs, which in turn contribute with the necessary infrastructure and faculty training on how to use the technology.

The relationship between infrastructure (i.e., and academic achievement in Brazilian large-scale assessments) has been extensively investigated. Research in developed countries indicate that infrastructure does not have a significant effect on the academic achievement (see Sammons, 1995, and OECD, 2014b for example). According to studies by Velez et al. (1994), Espósito et al. (2000), Barbosa et al. (2001), van Batenburg and Laros (2002), Biondi and Freitas (2007), Franco et al. (2007), Murillo (2007), Murillo and Román (2011), and Paget et al. (2016), only a modest relationship was found between these two variables. Another important study carried out by OECD (2014b) on the Programme for International Student Assessment (PISA) found only a weak relationship between educational resources and student achievement. In this study, there was more variance explained by the quality of human resources than by material and financial resources, similar to results found among industrialized nations. Albernaz et al. (2002) also stated from their findings that the more qualified the teachers are, the better the general results for academic achievement.

School public areas also highly significant for both Portuguese and Mathematics [$\chi_{\left(M\right)}^{2}$ = 25.83, *p* < 0.001; $\chi_{\left(P\right)}^{2}$ = 28.18, *p* < 0.001]. This variable measures the presence of laboratories, libraries, playgrounds, cafeterias, and sports courts. This finding is consistent with the studies conducted by Franco and Bonamino (2005), Bezerra and Kassouf (2006), Marzocchi and Oliveira (2009), Neji et al. (2014), and Paget et al. (2016) who agree that laboratory facilities, libraries, and audiovisual materials had a substantial effect on school achievement. However, although school infrastructure can play a significant role in improving the quality of education at large, Franco and Bonamino (2005) highlight that these resources themselves are not sufficient to ensure the increase of student achievement, as this occurs due to the interaction of different factors and it depends on whether they are effectively and consistently used by students. Further, Hanushek (2005) suggests that the effect of infrastructure and resources on achievement varies with the level present across the educational system.

Public policies that have a positive impact in the learning capabilities of the students are necessary to increase school achievement. According to Soares and Alves (2003), factors that impact cognitive achievement can be categorized into four groups: pedagogical projects, school structure, family, and personal aspects of the student.

## Conclusion

Findings presented in this study may contribute to identifying which variables are most influential on Brazilian student's achievement and therefore may serve as a guide in future decisions about educational policies. *Prova Brasil* aims to provide information on students' educational achievement across different stages of their development and how several socioeconomic variables are related to their performance. The variables examined in this study were addressed in a three level model (student, class, and school) in order to reflect the within school complexities of educational interactions—a common critique of school effectiveness studies that do not employ this methodology (Goldstein and Woodhouse, 2000). Additionally, the relatively large number of factors examined in this study further addresses critiques that there is little evidence to support simplified and short lists of factors that contribute to educational achievement and school effectiveness (Goldstein and Woodhouse, 2000).

It should be noted that the use of standardized tests themselves have a wider implication for education, appropriate teaching, and all relevant policies. Such arguments are naturally highly valuable and relevant, and should be considered alongside any and all arguments made in this body of work. The limitations of large scale testing data are numerous and have been addressed extensively in the literature. Critiques range from the limitations of the use of large scale multiple choice testing as a measurement of educational quality or achievement (see Sayed and Kanjee, 2013 for example), to the limitations of the predictor variables themselves (see Wiliam, 2008 for example), and of course the over-simplification of the complex and dynamic processes that characterize education and learning—a limitation acknowledged even by the foundational researchers into school effectiveness factors (see Becker, 1964; Harbison and Hanushek, 1992 for example). However, for the purposes of the research and recommendations made here, arguments focus on the idea of approaching policy utilizing an increasingly-used analytical technique that could be applied to any measure of performance (or comparable outcome measure of interest). As such, we state that this piece does not seek to argue for or against the use of *Prova Brasil* itself as a sufficient indicator, but rather, assumes its use as one.

In summary, the significant variables of the first level cluster around two main groups. The first one concerns the time available or dedicated to studies. As a general trend, more time dedicated to other activities not directly related to schoolwork has a negative association with achievement scores, and the opposite is also observed. The second cluster is concerned with the influence of parents in the student's academic life. The positive influence can be either direct through parental involvement in homework or indirect, through the level of education of parents, for instance. The second level variables clustered around the conditions of the teacher's working environment and also around their qualifications. In general, more qualification and better working conditions correlate positively with *Prova Brasil* scores. The third level variables are mostly associated with the quality of school infrastructure, in which positive trends are surprisingly not ubiquitous (e.g., Didactic Materials). These results both refine and advance the current research on school effectiveness studies in the developing world. While they find some basis in international trends (see Scheerens, 2000, 2001), this study represents one of a very limited number of English language studies using Prova Brasil data to examine important factors of progression for Brazilian students (see Paget et al., 2016 for example). This study, therefore, permits a broader comparison between Brazilian data and other large scale international studies (see Segretin et al., 2009 for example).

The results are presented by level due to the type of statistical technique employed in this study, i.e., Multilevel Modeling. The critical benefit in using the MLM approach in Brazil is its ability to present critical findings across the complex and many-dimensional domains relevant to education in a large and diverse population. When applied to educational testing, MLM produces relevant levers for policymakers. Utilizing levers is only possible when understanding why any particular finding may have occurred as well as comparator results for establishing priority. As such, the level-based analysis is particularly vital for identifying potential solutions out of a range of possible options, and establishing clear targets for improvement, which has clearly provided impetus for schools in Brazil.

Despite considerable investment and restructuring of the Brazilian educational system over the last 20 years, Brazil continues to lag behind its OECD counterparts on international educational ranking systems (Bruns et al., 2012). According to 2012 PISA reports, education in Brazil scored among the 10 lowest in both mathematics and reading (PISA, 2012). Through the continuous monitoring and analysis of Prova Brasil data, the Brazilian Ministry of Education is in a position to target future policies and funding where it may be best used in order to address continued deficiencies. The results of this study may help direct the Ministry's focus to some of the most important variables of student achievement. This, of course, is not a fool-proof system. For example, Silva et al. (1993) state that the reason behind Brazil's low educational achievement might be closely related to the lack of investments in educational structure, observed, for instance, in high budget programs that lack organization and proper control from authorities. These programs are then discontinued for not achieving the expected goals or become somewhat demoralized for the same reasons. At the very least, educational policies can be difficult to formulate and implement given the dynamic, complex, and differentiated settings in which teaching and learning takes place (Teddlie, 1994; Teddlie et al., 2000). The significance of large-scale analyses such as the one conducted in this study, however, can offer directions for further study with more sensitive instruments.

A few limitations must be addressed from the outputs of this research. Firstly, even though there is a nationwide curriculum that is expected to be followed by every Brazilian School, no adaptations or statistical techniques (e.g., Differential Item Functioning) were utilized to compensate or identify occasional differences in how subjects are taught to students.

Another relevant structural limitation concerns the absence of variables that seek to assess individual characteristics on a personal level. According to Soares and Alves (2003), personal aspects of the students impact cognitive abilities. Our concern lies on the underrepresentation of this group of variables on the socioeconomic questionnaires of Prova Brasil. Several authors argue that the low amounts of explained variance in studies in the field of educational are related to the lack of data on individual characteristics, such as cognitive abilities and other personality related features as motivation and persistency (Teddlie and Reynolds, 2000; Soares et al., 2001).

As this study addresses a wide range of variables that influence academic achievement from a broader perspective, only random intercept models were utilized for achievement prediction. Random slope and multilevel models with interactions between variables were therefore not considered. Moreover, only the overall scores for the whole of the country were taken into account, but not scores by regions or states. Considering these limitations, future research exploring further relations among variables from different levels and focused on states and regions is recommended.

## Author contributions

As the first author, IM contributed to organize and write all the sections of the paper. SS contributed to organize the literature review, discussion, and conclusion. VD collaborated to carry out the data analysis and to describe the results. TV and EF collaborated to write the results and discussion. CP as a specialist in educational policies was responsible to review the whole of the paper and to contribute to the discussion and conclusions. KR was responsible to discuss educational policies in light of his experience on this topic and contributed to the whole of the paper.

### Conflict of interest statement

The authors declare that the research was conducted in the absence of any commercial or financial relationships that could be construed as a potential conflict of interest.
